# Artificial intelligence in preoperative diagnosis of thyroid nodules: a bibliometric analysis

**DOI:** 10.3389/fonc.2026.1860859

**Published:** 2026-08-12

**Authors:** Weiqi Wu, Yuru Xu, Hailong Wang, Yan Han, Youjun Liu, Yifeng Chen

**Affiliations:** 1 Department of Pathology, Quanzhou First Hospital Affiliated to Fujian Medical University, Quanzhou, China; 2 The School of Basic Medical Sciences, Fujian Medical University, Fuzhou, China

**Keywords:** artificial intelligence, bibliometric analysis, fine needle aspiration cytology, thyroid nodules, ultrasound

## Abstract

**Objectives:**

To explore the current research progress and trends in the application of artificial intelligence (AI) for preoperative diagnosis of thyroid nodules (TN), and to identify the key research directions through bibliometric analysis.

**Methods:**

Articles on the application of AI for preoperative diagnosis of TN were retrieved from the Web of Science core collection and Scopus databases. Tools like VOSviewer, CiteSpace, the bibliometrix, Scimago Graphica, and Charticulator were used for bibliometric analysis. Trends in annual publishing volumes, collaborations between authors and institutions, highly cited articles, keyword co-occurrence, keyword clustering, and keyword burst analysis are all included.

**Results:**

A total of 1370 publications were included in this study, with an rapid growth trend observed in the annual publications in this field. China has made significant contributions, with Shanghai Jiao Tong University producing the most publications. Chinese scholar XuDong emerged as the most prolific author (30 publications). Journal analysis revealed that *Thyroid* is the highest-impact journal in terms of citation frequency. High-frequency keywords in the field include “TN”, “cancer” and “ultrasound”, while recent emerging keywords include “semantic segmentation”, and “AI”.

**Conclusions:**

Since 2019, the application of AI in the preoperative diagnosis of TN has attracted increasing research attention, with China emerging as a major contributor to this research field. The number of journals publishing in this domain has significantly increased, and the publications reflect a trend toward interdisciplinary research. The research focus has gradually shifted from conventional diagnostic approaches toward pixel-level lesion quantification, fine-grained feature extraction and the integration of AI-enabled diagnostic approaches, highlighting the potential of AI to improve TN assessment.

## 1 Introduction

Recent advancements in ultrasound imaging technology, coupled with an increased public awareness of health screening, have significantly elevated the detection rate of thyroid nodules (TN) in the population. Currently, the detection rate of TN using high-resolution ultrasound ranges from 20% to 76%, and 8% to 16% of these cases are malignant (1). According to GLOBOCAN figures, around 821,000 new instances of thyroid cancer were reported globally in 2022, making it the seventh most frequent cancer, with a much greater prevalence in females compared to males (2).

Currently, the evaluation of TN comprises four primary steps: clinical examination, thyroid function testing, ultrasound, and fine needle aspiration cytology (FNAC) (3). Ultrasound is the chosen initial diagnostic technique in clinical practice because of its excellent sensitivity and specificity for TN evaluation (4). However, ultrasound interpretation is subjective, influenced by factors such as physician experience and imaging equipment resolution, which can result in both false negatives and misdiagnoses, ultimately impacting treatment decisions (5). Notably, FNAC remains the gold standard for preoperative pathological diagnosis of TN, but the Bethesda System’s classification of uncertain cytological categories presents a core challenge in clinical diagnosis and treatment (6). Its accuracy is significantly influenced by both procedural technique and the clinician’s diagnostic experience. This limitation is especially apparent in indeterminate categories, such as “atypia of undetermined significance/follicular lesions of undetermined significance” and “suspicious for follicular neoplasm”, which frequently pose diagnostic challenges and may necessitate repeat biopsies or unnecessary surgeries (7). In addition, the malignancy risk of partially cystic TN ranges from 3.3% to 17.5%. FNAC specimens from these lesions frequently contain scant follicular epithelial cells, predisposing to false-negative cytology and thereby increasing the difficulty of diagnostic interpretation (8). Consequently, it places higher demands on the expertise of both the performing clinician and the cytopathologist.

With the exponential growth of healthcare big data, artificial intelligence(AI) has created numerous opportunities in medical diagnosis. AI is capable of automatically detecting and assessing complex medical images, leading to more accurate and efficient diagnoses (9). AI-based ultrasound imaging and cytopathological diagnoses of TN can minimize physician burden while improving diagnostic precision (10, 11).

Systematic reviews play many critical roles. They can synthesize existing research findings in a field, clarify the research context, and further identify priorities for future research (12). The literature search, or information retrieval process, constitutes a core step in conducting systematic reviews. It not only affects the outcomes of systematic reviews but also serves as the fundamental procedure for establishing analyzable datasets (13). Bibliometrics, a field that uses mathematical and statistical methods to quantify published books and journal articles, has emerged as a valuable tool for visualizing and analyzing key information in specific research domains, such as authors, institutions, countries, citations, and keywords (14). It aids in literature synthesis by providing significant insights into a field’s development trends and research hotspots (15).

The use of AI in the preoperative identification of TN is fast increasing. However, rigorous bibliometric assessments that thoroughly map the worldwide research scene, detect new patterns, and project future directions in this discipline are still very rare. This work uses bibliometric approaches to thoroughly examine relevant articles from 2015 to 2026, with the goal of capturing the current state, recent advances, and frontier research hotspots in this subject, as well as indicating potential future research directions.

## 2 Materials and methods

### 2.1 Data collection

Our study selected the Web of Science Core Collection (WoSCC) and Scopus databases for literature retrieval. All searches were completed on the same day to preclude potential bias caused by daily database updates.

### 2.2 Search strategy

To ensure that the search results were highly relevant and comprehensive to the topic of the application of AI in the preoperative diagnosis of TN, the final search query was determined after referencing the Medical Subject Headings (16) and other specialized terms. For the WoS database, the search query was formulated as follows: TS=((“artificial intelligence” OR “machine learning” OR “deep learning” OR “feature learning” OR “neural network” OR “bayesian network” OR “Supervised Learning” OR “Unsupervised Learning” OR “computer-aideddiagnosis” OR “CADx” OR “digital pathology” OR “multimodal” OR “Iargelanguage models”) AND (“cytology” OR “fine needle aspiration” OR “ultrasound” OR “ultrasonography” OR “radiomics” OR “TI-RADS” OR “Bethesda” OR “cytopathology images” OR “elastography”) AND (“thyroidnodule” OR”thyroid nodules” OR “thyroid neoplasm” OR “thyroid tumor” OR “thyroid mass” OR “thyroid lesion”)). In the Scopus database, the search strategy used was: (TITLE-ABS-KEY (“artificial intelligence” OR “machine learning” OR “deeplearning” OR “feature learning” OR “neural network” OR “bayesian network” OR “Supervised Learning” OR “Unsupervised Learning” OR “computer-aideddiagnosis” OR “CADx” OR “digital pathology” OR “multimodal” OR “largelanguage models”) AND TITLE-ABS-KEY (“cytology” OR “fine needleaspiration” OR “ultrasound” OR “ultrasonography” OR “radiomics” OR “TI-RADS” OR “Bethesda” OR “cytopathology images” OR “elastography”) AND TITLE-ABS-KEY (“thyroid nodule” OR “thyroid nodules” OR “thyroidneoplasm” OR “thyroid tumor” OR “thyroid mass” OR “thyroid lesion”). The retrieval was restricted to English-language articles published between January 1, 2015, and July 8, 2026.

### 2.3 Literature screening

A systematic literature search was conducted in the WoS and Scopus databases. Initially, 1227 publications were retrieved from WoS, with “all records and cited references” exported in plain text format. 1802 publications were retrieved from Scopus, with all search records exported in CSV format. These CSV files were converted into plain text format using Python for data unification. To ensure comprehensiveness, specific inclusion and exclusion criteria were applied.

#### 2.3.1 Inclusion criteria

Studies must address the application of AI in the preoperative diagnosis of TN; only English-language original articles were included; all publications were published in academic journals.

#### 2.3.2 Exclusion criteria

Reviews, comments, meeting abstracts, letters, as well as studies with incomplete information or duplicate records were excluded.

#### 2.3.3 Removal of duplicate records

Records were retrieved from the WoS and Scopus databases, and cross-database duplicate records existed in the retrieved results. Python programming was used in this study to perform automatic deduplication. Duplicate records were identified and removed by comparing metadata including literature DOIs, titles, and author information.

#### 2.3.4 Literature screening process

**Figure 1:**
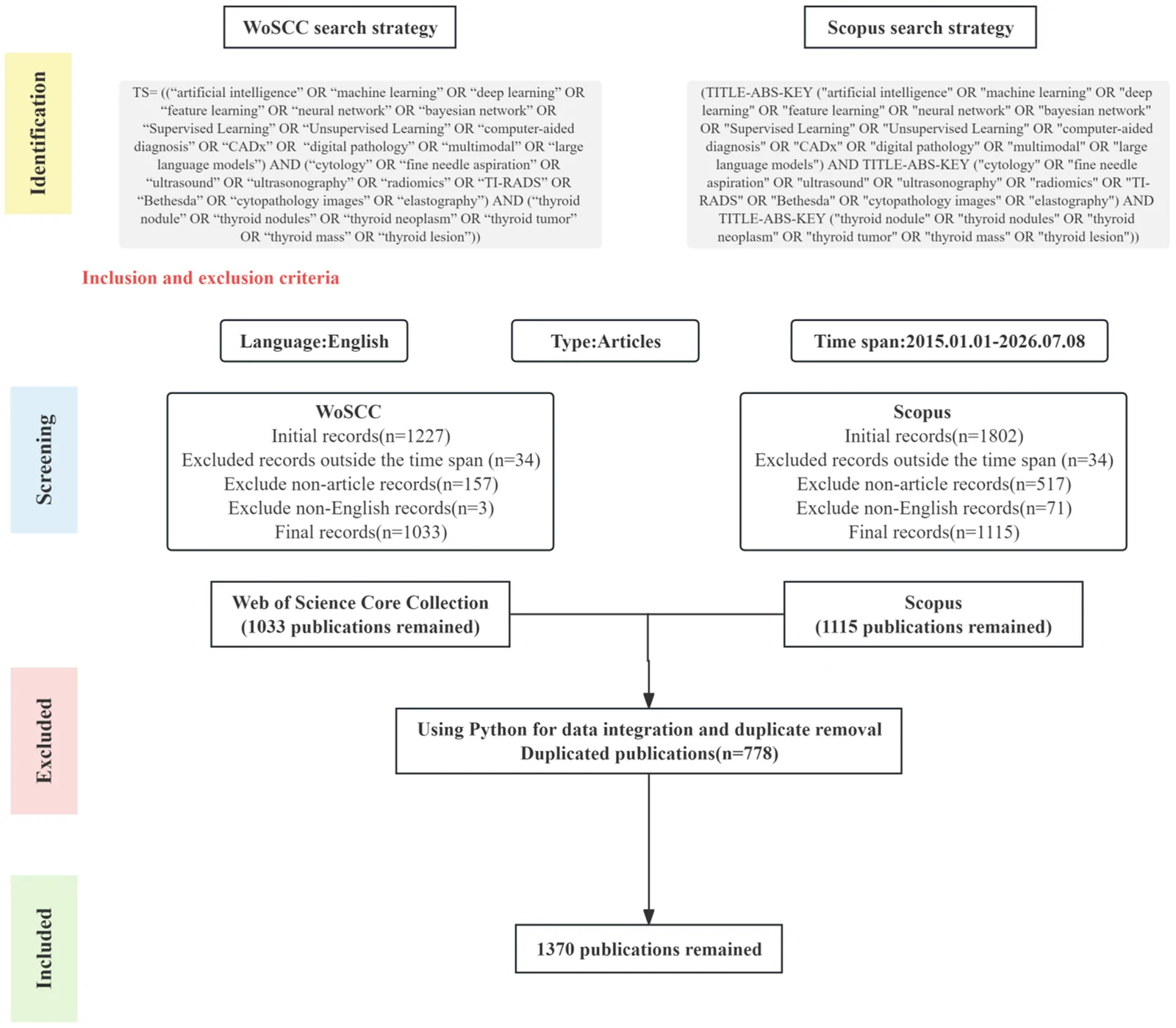
The retrieval strategy and data analysis process of this study.

The literature screening was followed the core principles of the PRISMA 2020 guideline (12) to ensure methodological rigor. Two researchers independently conducted the primary and secondary screening and assessed the relevance of records. Any disagreements were resolved through discussion together with the corresponding author until consensus was achieved. The procedures of literature identification, screening and selection are presented in **Figure 1**, and a total of 1370 publications met the inclusion criteria finally.

### 2.4 Data standardization

This study conducted standardization processing on country/region names, institutions, author and keywords. For country/region names: (i) Regional entries including England, Scotland, Wales and Northern Ireland were harmonized as the United Kingdom; (ii) Spelling variants such as Türkiye and turkiye were standardized to Turkey; (iii) Country names were substituted by internationally recognized abbreviations, for instance, “United States” was replaced with “USA”. Institutional names were standardized using Python that systematically identified and consolidated variant representations—including official translations, standardized abbreviations, and aliases—of the same organization. For authors, Python helped standardize signatures; variants including “Dong Xu”, “Xu D” and “D Xu” were unified into the format of “surname + initial” (e.g., “Xu D”). For keywords, manual cleaning harmonized singular/plural forms and synonyms. Specifically, this included merging “thyroid nodule” and “thyroid nodules” into “thyroid nodules”. Non-specific, context-independent terms such as “na”, “human”, and “article” were excluded to prevent interference with core topic analysis. All data standardization work was independently completed by two researchers, and discrepancies were settled through group discussion.

### 2.5 Bibliometric methods and visual analysis

Various tools and software were employed to conduct bibliometric analysis. These tools were used for the visual analysis of countries, institutions, authors, journals, co-cited papers, and keywords. A brief overview of the tools and their applications in this study is provided below.

Scimago Graphica (version 1.0.55; Scimago Lab): The GML-formatted collaboration network file exported from VOSviewer was imported into the software. A global geographic distribution map of international research collaboration was constructed by integrating the network data with standardized geographic coordinate information (parameter settings: label=Country, cluster=String). Concurrently, an institutional collaboration network was built using institutional collaboration data (parameter settings: label=String). Node size represented publication volume; edge width reflected the frequency or strength of collaborative ties; node colors distinguished different clusters, and node color was used to map connection strength. Default configurations of the software were adopted for layout, layer filtering and other visualization parameters.

Charticulator (Microsoft Research): The node attribute statistical table exported from Gephi was imported to generate a chord diagram of international research collaboration. The length of each arc represented the publication output of each country, and the thickness of connecting lines reflected the intensity of bilateral research collaboration. All visualization parameters—including layout configuration, color assignment, and scaling—were set to Charticulator’s default settings.

VOSviewer (version 1.6.20; Leiden University Centre for Science and Technology Studies) was used to construct the author collaboration network and journal co-occurrence density map. Full counting was employed, and association strength was selected as the normalization method. For the author collaboration network, layout attraction=2 and repulsion=1; for the journal co-occurrence density map, layout attraction= 4 and repulsion=−2.Clustering resolution of both maps was set to 1.00, minimum cluster size=1, and merging small clusters was enabled. Documents (publication count) were chosen as the weight indicator for visualization. The author collaboration network used software-automatic link thresholds based on minimum association strength with colored curved lines enabled, and item density was utilized for journal density mapping. Node size denoted publication volume, link thickness reflected collaboration tightness, and color gradients differentiated clusters and node aggregation levels. The author collaboration network was exported as JSON and imported into vosviewer.vip (https://www.vosviewer.vip) to plot the author collaboration chord diagram.

CiteSpace (version 6.4.R1; Chen Chaomei) was used to build the keyword co-occurrence clustering network and the keyword burst detection map. The specific parameters were configured as follows: time slicing spanned from 2015 to 2026 with a slice length of one year; keyword selection followed the g-index criterion (k = 25), with LRF = 2.5, L/N = 10, LBY = 5, and e = 1.0; no pruning strategy was performed). For burst detection, the parameters were set to α1/α0 = 2.0, αi/αi-1 = 2.0, number of states = 2, γ=1.0, and minimum burst duration = 2. The top 25 keywords ranked by burst strength were visualized. Node size represented keyword frequency, edge thickness reflected the strength of co-occurrence between keywords, and distinct colors corresponded to different thematic clusters. Betweenness centrality was employed to identify pivotal hub keywords that served as bridging nodes in the network.

Bibliometrix (Dr. Massimo Aria): an integrated bibliometric toolkit based on the R language. In this study, Bibliometrix was used to perform bibliometric statistical analysis, including identifying core journals in accordance with Bradford’s law, analyzing the annual publication output of the top 10 journals, and generating keyword word clouds. Default computational parameters of the toolkit were adopted for all analyses.

## 3 Results

### 3.1 Database search

A total of 1370 articles were retrieved from the WoS and Scopus databases. Research pertaining to this field was published by scholars from 68 countries/regions, with 6576 researchers from 1905 institutions participating. The studies were published in 432 different journals, and the literature encompassed 2580 distinct keywords.

### 3.2 Annual publications, citations and trends

**Figure 2:**
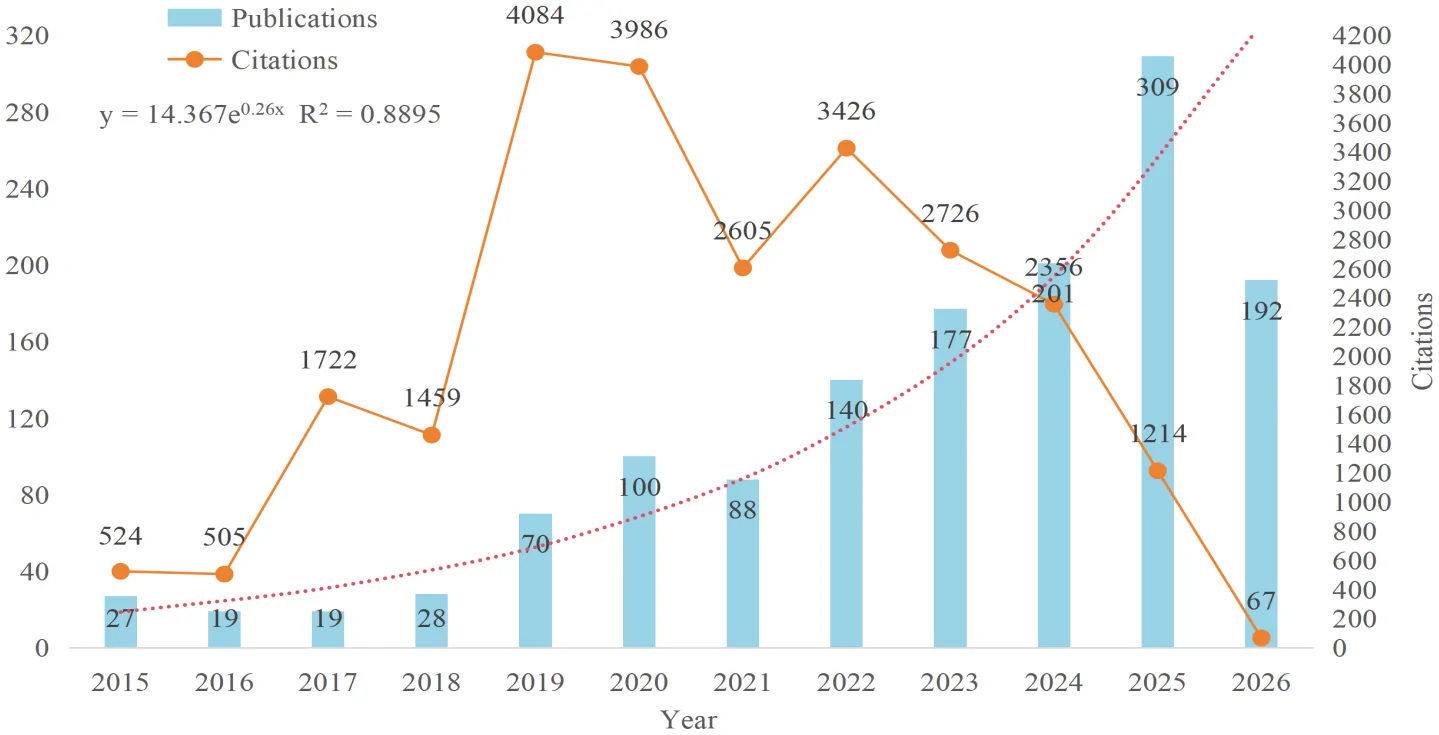
The annual publication and citation trends of AI in preoperative diagnosis of TN between 2015 to 2026.

As shown in **Figure 2**, the number of annual publications on AI in preoperative diagnosis of TN has demonstrated an rapid growth trend from 2015 to 2026. During the early years(2015-2018), the publications remained relatively low with modest growth. In recent years, particularly since 2019, there has been a substantial increase in both publications and citations. It indicates that there was a substantial progress in 2019 and laid the foundation for subsequent research in the field. In conclusion, the research prospects of AI in the field of preoperative diagnosis of TN are promising, with considerable potential for further growth.

### 3.3 Countries/regions

**Figure 3:**
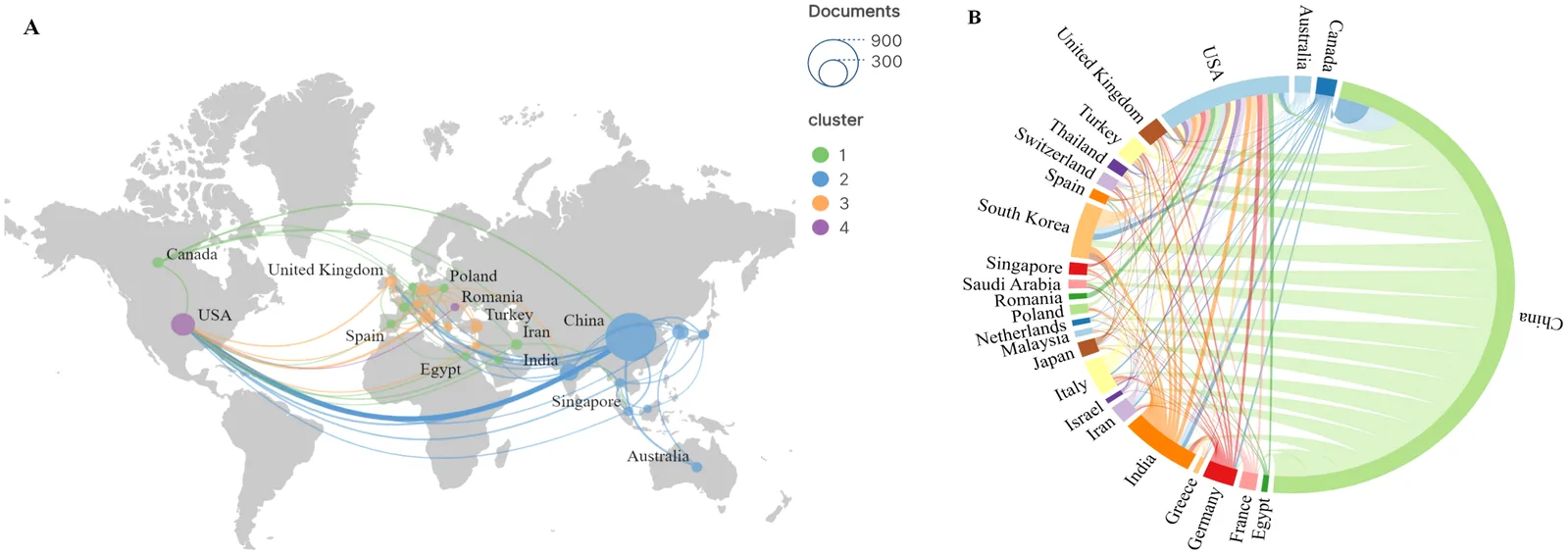
Analysis of countries/regions in the field of AI in preoperative diagnosis of TN. **(A)** The geographic visualization map generated by using Scimago Graphica. **(B)** The international cooperation map created with Charticulator.

Among the retrieved literature, a total of 68 countries have contributed to research in this field; the top 10 countries ranked by the number of publications listed in **Table 1**. China (851 publications, 13879 citations) and the USA (174 publications, 6125 citations) are the top two countries dominate both publications and total citations, accounting for 74.82% of the total number of articles. The centrality values for these two countries are 0.50 and 0.39, respectively. As depicted in **Figures 3A, B**, the national collaboration network reveals that the most widely connected countries are China and the USA. It indicates that China and the USA are the driving forces in the field and serve as the key hubs of the international cooperation network. Additionally, the Canada and USA lead in terms of average citations per paper, with 38.62 and 35.20 citations, respectively.

**Table 1:** The top 10 countries/regions by number of publications on AI in preoperative diagnosis of TN.

Rank	Country	Publications	Citations	Centrality
1	China	851	13879	0.50
2	USA	174	6125	0.39
3	India	97	1868	0.17
4	South Korea	77	2641	0.02
5	Italy	50	1322	0.14
6	Germany	41	702	0.10
7	United Kingdom	33	631	0.09
8	Turkey	32	316	0.01
9	Canada	26	1004	0.02
10	Iran	25	257	0.03

### 3.4 Institutions

**Figure 4:**
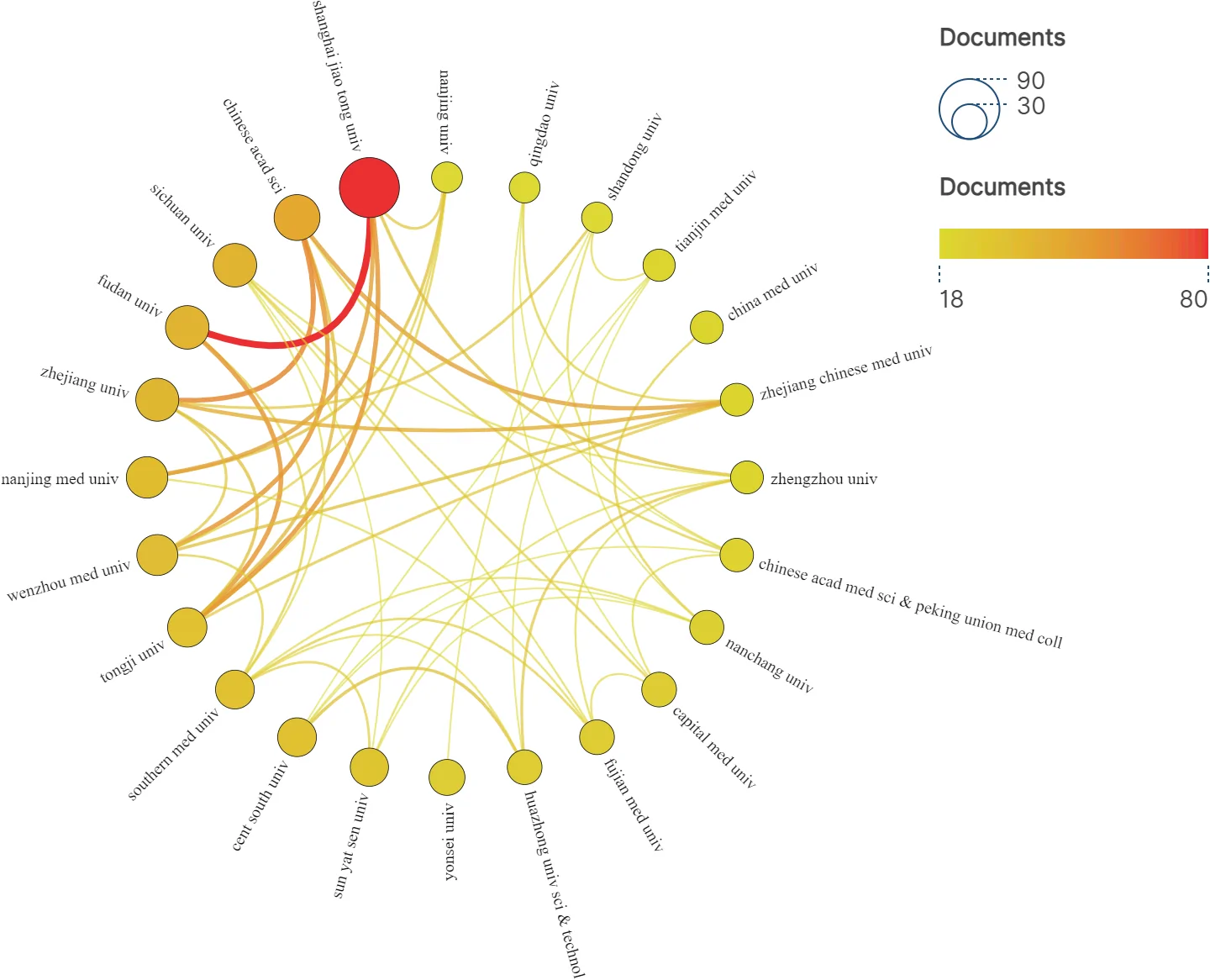
Collaborative networks among the top 24 most productive institutions using Scimago Graphica.

From 2015 to 2026, a total of 1905 research institutions involved in this field published relevant works. Among them, the top 10 institutions (**Table 2**) predominantly based in China. Shanghai Jiao Tong University emerged as the most prolific, publishing 80 papers and receiving a substantial citation count of 2110, which far exceeded that of other institutions. Following closely are Chinese Academy of Sciences (45 papers) and Fudan University (40 papers). Furthermore, Southern Medical University and Fudan University ranked highest in terms of citations per paper, with averages of 41.52 and 33.53, respectively. Evidently, Chinese research institutions are the key contributors to advancements in this field. The analysis of the collaborative network among the top 24 institutions by publication output (**Figure 4**) reveals an intensive relationships of cooperation among leading institutions in the field. In the figure, node size and color intensity correspond to publications, while the thickness of the connecting lines reflects the strength of collaboration between institutions.

**Table 2:** The top 10 most productive institutions.

Rank	Institution	Country	Publications	Citations
1	Shanghai Jiao Tong University	China	80	2110
2	Chinese Academy of Sciences	China	45	943
3	Fudan University	China	40	1341
4	Sichuan University	China	40	951
5	Zhejiang University	China	39	1001
6	Nanjing Medical University	China	36	454
7	Wenzhou Medical University	China	35	658
8	Tongji University	China	32	639
9	Southern Medical University	China	31	1287
10	Central South University	China	31	343

### 3.5 Authors

**Figure 5:**
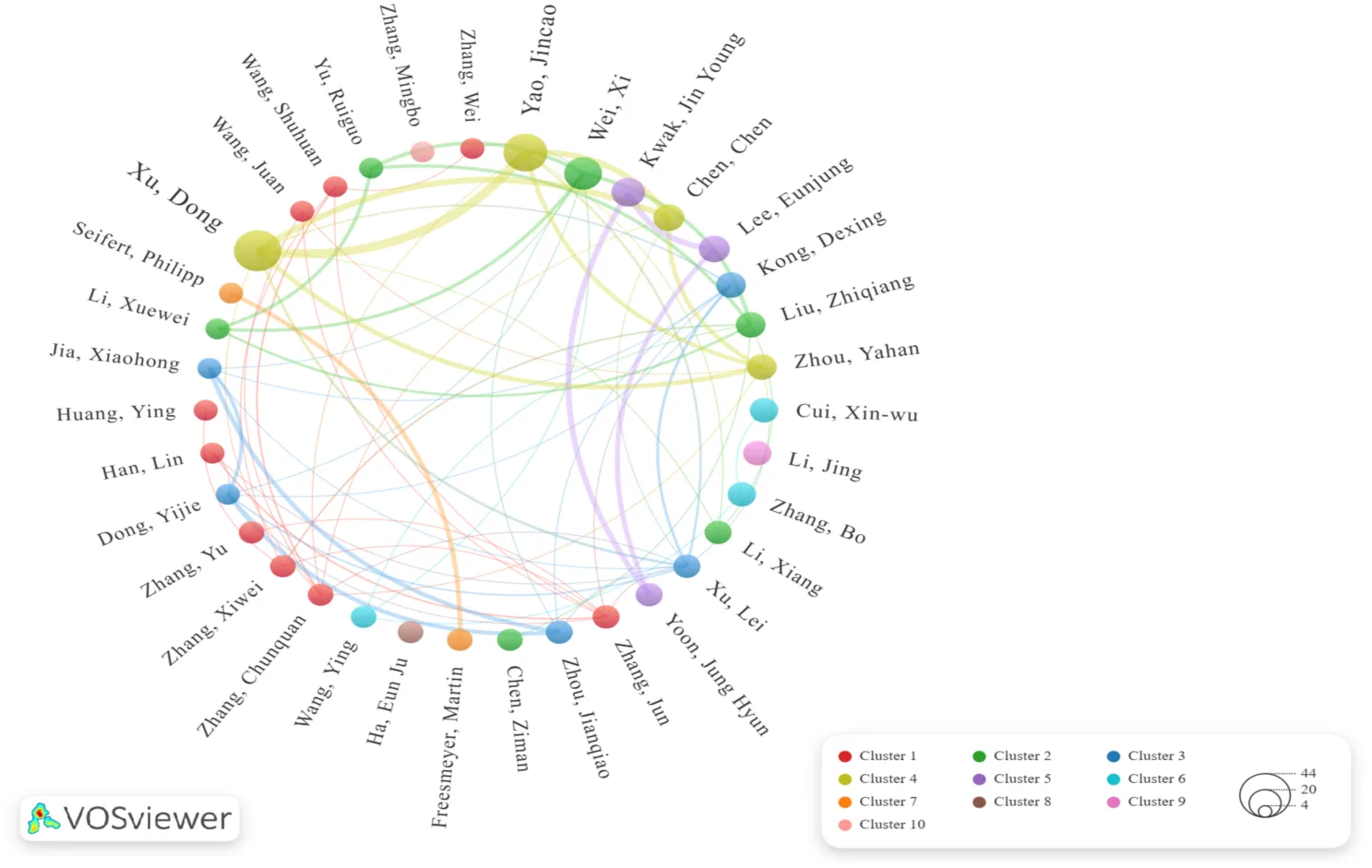
The collaborations’ visualization map of authors. (different colors indicate distinct research clusters).

A total of 6576 authors participated in the publication of 1370 articles in the field of AI in preoperative diagnosis of TN. **Table 3** presents the top 10 most productive authors in this area. It can be intuitively seen that Korean scholars stand out with remarkable H-index scores, while Chinese authors, leading in publications, exhibit substantial variation in their H-index distribution. The H-index, which combines both the number of publications and citations, offers a more meaningful measure of academic reputation and influence compared to simple publications or citations count (17). The top authors with the highest H-index were Kwak Jin Young (51). As illustrated in **Figure 5**, researchers in this field exhibit strong collaborative relationships both within and across different clusters. Among the most prolific contributors are Xu Dong (30 publications) and Yao Jincao (26 publications). Notably, the research cluster led by Xu Dong and Yao Jincao has made significant contributions to the field, with their publications demonstrating a high degree of collaboration among authors. Affiliated with Zhejiang Cancer Hospital, their work primarily focuses on the application of deep learning algorithms in addressing the TN problem, making significant contributions to advancements in cancer diagnosis and treatment.

**Table 3:** The top 10 authors by publication output in AI in preoperative diagnosis of TN.

Rank	Author	Country	Publications	Citations	H-index
1	Xu Dong	China	30	598	19
2	Yao Jincao	China	26	320	13
3	Wei Xi	China	20	839	28
4	Kwak JinYoung	South Korea	16	409	51
5	Chen Chen	China	14	139	3
6	Lee Eunjung	South Korea	14	299	21
7	Zhou Yahan	China	13	71	28
8	Liu Zhiqiang	China	13	315	15
9	Kong Dexing	China	13	665	28
10	Zhang Bo	China	12	355	15

### 3.6 Journals

**Figure 6:**
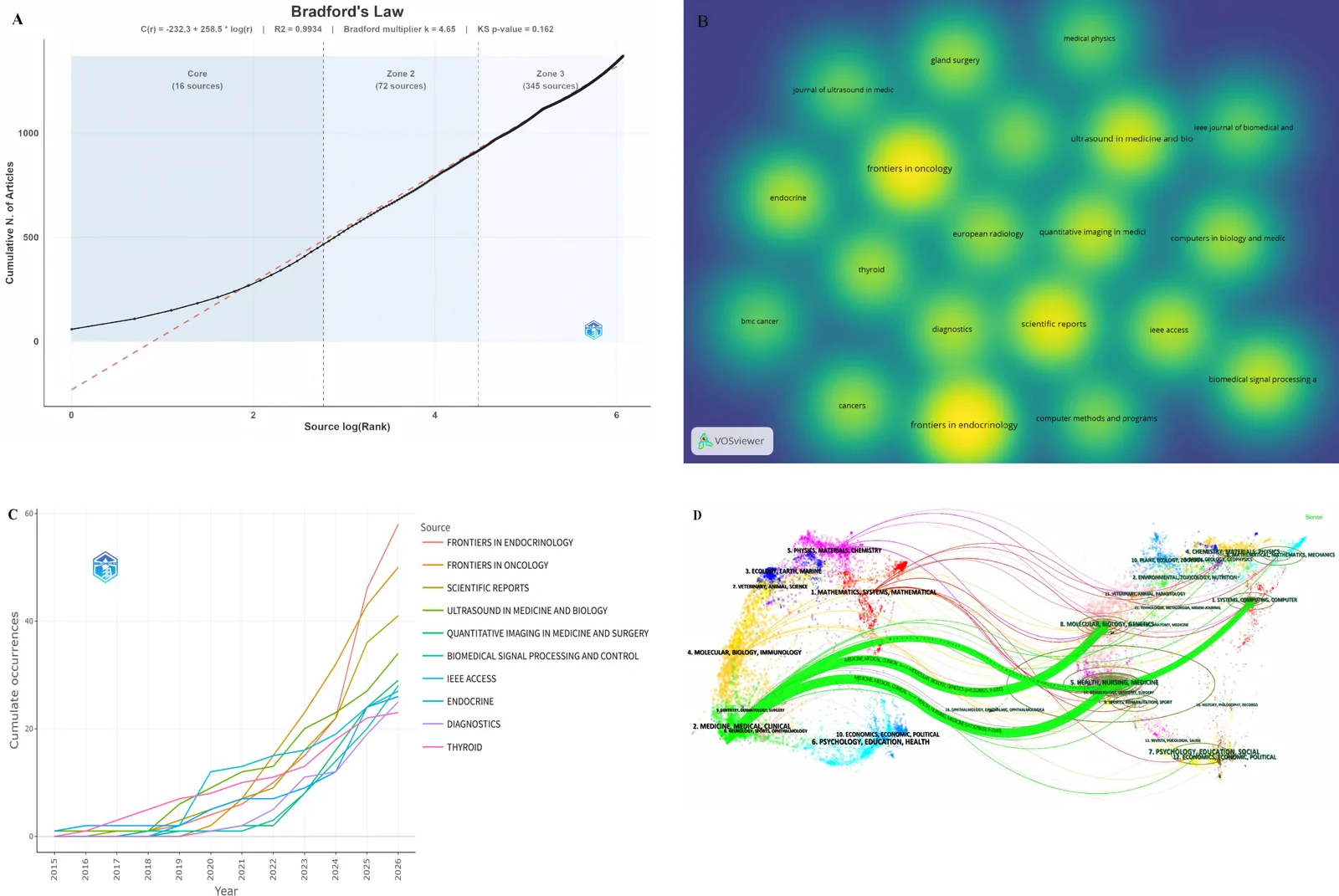
Analysis of journals in the domain of AI in preoperative diagnosis of TN. **(A)** The Core sources by Bradford’s law. **(B)** The map of journals with ≥ 15 publications. **(C)** Trend of the number of articles in the top 10 most productive journals. **(D)** The dual-map overlay of journals.

The literature included in this study was published in 432 different journals. Bradford’s Law can be used to identify core journals in a specific field (18). As shown in **Figure 6A**, an analysis based on Bradford’s Law in Bibliometrix identified 16 core source journals with 466 articles, accounting for 34.01% of the total number of articles. As shown in **Table 4**, among these core journals, 4 were JCR Q1 journals (with 2 journals having an impact factor (IF) greater than 5), 5 were Q2 journals, and 1 was a Q3 journal. Geographically, the USA, Switzerland, and United Kingdom each contributed 3 core journals. **Figure 6B** shows the 20 journals that published 15 or more papers, with node color representing the number of publications; the larger the node, the brighter yellow the number of publications. *Frontiers in Endocrinology* had the highest publication volume (58 articles, IF: 5.7), followed by *Frontiers in Oncology*, which published 50 articles. *Thyroid* had the highest IF(6.6) and total citation count (853 citations), but only published 23 articles. As shown in **Figure 6C**, starting from 2023, *Frontiers in Oncology* and *Frontiers in Endocrinology* became the leading journal in terms of publication volume, and the publication output of other journals also increased during this period. This marked a phase of rapid development in the research on AI in the preoperative diagnosis of TN.

**Table 4:** The top 10 journals ranked by publication output.

Rank	Journal	Country	Publications	IF	JCR
1	Frontiers in Endocrinology	Switzerland	58	5.7	Q1
2	Frontiers in Oncology	Switzerland	50	3.4	Q2
3	Scientific Reports	United Kingdom	41	4.9	Q1
4	Ultrasound in Medicine and Biology	United Kingdom	34	2.8	Q2
5	Quantitative Imaging in Medicine and Surgery	China	29	2.8	Q2
6	Biomedical signal processing and control	United Kingdom	28	5.7	Q2
7	Ieee access	USA	27	4.2	Q2
8	Endocrine	USA	26	3.3	Q3
9	Diagnostics	Switzerland	25	3.8	Q1
10	Thyroid	USA	23	6.6	Q1

Journal dual-map overlay analysis helps to understand the development trajectory of a discipline and track frontier trends (19). The left side of the distribution represents the citing journals, reflecting the current research landscape in the relevant field, while the right side represents the cited journals, indicating the foundational research. The citation relationships between the two are displayed as colored curved bars from left to right. **Figure 6D** presents the results of the dual-map overlay visualization analysis of journals in the field of AI in the preoperative diagnosis of TN. Three main citation paths were identified. The citing journals predominantly belong to medicine, medical, and clinical journals, while the cited journals are mainly published in molecular biology, genetics, health, nursing, medical, and systems/computing/computer science journals..

### 3.7 Highly cited studies

**Table 5** lists the top 10 most cited papers, all of which have been cited more than 200 times. The paper published in Lancet Oncology in 2019, titled ‘Diagnosis of thyroid cancer using deep convolutional neural network models applied to sonographic images: a retrospective, multicohort, diagnostic study,’ has the highest citation count (409 citations) (20). The second most cited paper is by ChiJN, which presents a computer-aided diagnostic system for TN classification based on ultrasound images (21). The third most cited paper is by Patel KN and his team, who proposed a genomic sequencing-based classification model to address the challenge of preoperative diagnosis of ‘indeterminate’ results in FNAC (22). This paper has garnered significant citations in the field.

**Table 5:** The top 10 most cited papers.

Rank	Title	Journal	First author	Total citations	Year
1	Diagnosis of thyroid cancer using deep convolutional neural network models applied to sonographic images: a retrospective, multicohort, diagnostic study	Lancet Oncology	Li XC	409	2019
2	Thyroid Nodule Classification in Ultrasound Images by Fine-Tuning Deep Convolutional Neural Network	Journal of Digital Imaging	Chi JN	375	2017
3	Performance of a Genomic Sequencing Classifier for the Preoperative Diagnosis of Cytologically Indeterminate Thyroid Nodules	Jama Surgery	Patel KN	352	2018
4	Deep learning-based artificial intelligence model to assist thyroid nodule diagnosis and management: a multicenter diagnostic study	Lancet Digit Health	Peng S	331	2021
5	2020 Chinese guidelines for ultrasound malignancy risk stratification of thyroid nodules: the C-TIRADS	Endocrine	Zhou JQ	330	2020
6	RadImageNet: An Open Radiologic Deep Learning Research Dataset for Effective Transfer Learning	Radiology. Artificial intelligence	MEI X	329	2022
7	A Survey of Deep-Learning Applications in Ultrasound: Artificial Intelligence-Powered Ultrasound for Improving Clinical Workflow	Journal of the American College of Radiology	Akkus Z	304	2019
8	Lymph node metastasis prediction of papillary thyroid carcinoma based on transfer learning radiomics	Nature communications	Yu JH	280	2020
9	Deep convolutional neural network VGG-16 model for differential diagnosing of papillary thyroid carcinomas in cytological images: a pilot study	Journal of Cancer	Guari Q	278	2019
10	A pre-trained convolutional neural network based method for thyroid nodule diagnosis	Ultrasonics	Ma JL	256	2017

### 3.8 Keywords

**Figure 7:**
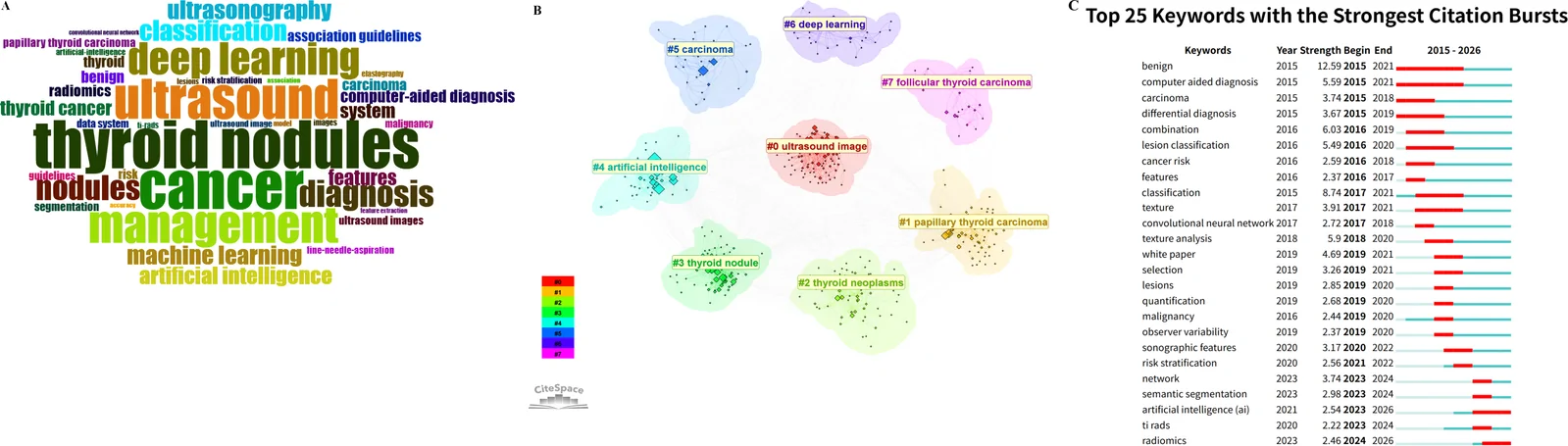
Analysis of keywords in the domain of AI in preoperative diagnosis of TN. **(A)** Word cloud of the top keywords. **(B)** The co-occurrence network of keywords clusters. **(C)** The top 25 keywords with the strongest citation bursts.

Frontier research hotspots and directions can be explored with the aid of keywords, which provide a concise description of study content. As shown in **Table 6**, “TN” (350 occurrences), “cancer” (334 occurrences), and “ultrasound” (292 occurrences) are the top 20 most frequent keywords (**Figure 7A**). Cluster analysis of the included keywords was performed to explore research hotspots in this field. The Q value and S value are used to evaluate the modularity and homogeneity of the cluster network. A Q > 0.3 identified the cluster structure as significant, and an S > 0.5 or > 0.7 indicated that the clustering result was reasonable or highly credible, respectively (23).As shown in the **Figure 7B**, these eight clustered nodes cover the application of AI in the preoperative diagnosis of TN, including keywords such as “ultrasound image”, “TN”, and “deep learning”. The cluster module value (Q = 0.4171) and average silhouette value (S = 0.7256) for the keywords indicate that these clusters are both significant and reasonable. Additionally, the **Figure 7C** illustrates the temporal evolution trends of the top 25 keywords with the highest burst strength. These keywords have seen a significant rise in frequency in a short period, serving as tools for rapidly identifying current research trends and discovering new research directions. The top 3 keywords with the highest burst strength are “benign”, “classification”, and “combination”. Keywords emerging after 2023 with significant burst strength include “network”, “semantic segmentation”, and “AI”.

**Table 6:** The top 20 most frequent keywords.

Rank	Keyword	Frequency	Rank	Keyword	Frequency
1	thyroid nodules	350	11	artificial intelligence	129
2	cancer	334	12	features	115
3	ultrasound	292	13	system	109
4	management	268	14	thyroid cancer	109
5	deep learning	237	15	computer-aided diagnosis	94
6	diagnosis	194	16	benign	88
7	nodules	183	17	radiomics	87
8	classification	152	18	carcinoma	85
9	machine learning	141	19	association guidelines	83
10	ultrasonography	140	20	thyroid	82

## 4 Discussion and conclusion

### 4.1 Discussion

Our study provides an in-depth analysis of literature published between 2015 and 2026 in the WoS and Scopus databases concerning the application of AI in the preoperative diagnosis of TN. Using visualization software, the study analyzes the research trends and hotspots in this field over the past decade. The objective is to help researchers extract valuable information from vast amounts of data, and to provide new research directions for clinicians and scholars.

In recent years, the number of publications in this field has shown rapid growth, particularly after 2019, when it entered an explosive phase. This indicates that the field has transitioned from an emerging area of exploration to a research hotspot. An analysis of high citations further validates this transformation: three of the top ten most cited papers were published in 2019. The research during this period laid the foundation for subsequent studies on algorithm optimization and multimodal data integration.

China and the USA are contributing to over 70% of global research output, making significant contributions to research on the application of AI in preoperative diagnosis of TN. This dominance reflects substantial investments in AI infrastructure in both countries. The top ten institutions in terms of publication volume are all based in China, with institutions such as Shanghai Jiao Tong University, the Chinese Academy of Sciences, and Fudan University showing a large output scale. This situation is closely related to China’s active promotion of the application of medical AI and its emphasis on fostering interdisciplinary talent (24). In addition to China, developed countries occupy a mainstream position in the field of AI for preoperative diagnosis of TN, despite the lower overall publication volume of the USA compared to China, its average citation per article ranks among the highest globally, alongside Canada, illustrating that its research outcomes possess significant academic impact and broad recognition. Looking ahead, cross-country collaboration is required to mitigate redundant expenditure and resource waste. Naturally, this goal needs to be realized through collaboration between relevant institutions and academic journals.

Professor Xudong from China ranks first in terms of the number of papers published in this field from 2015 to 2026, focusing on the development and application of intelligent diagnostic technologies. Xudong and his team developed the AI-assisted diagnostic system, the ThyGPT model, for the diagnosis and management of TN (25). Research shows that this model enhances the diagnostic capabilities of ultrasound physicians, assists in monitoring and identifying errors in reports, and ultimately improves the overall quality of diagnosis and treatment for TN patients.

Among the top ten authors ranked by publication volume, Professor Kwak JinYoung from South Korea ranks first with an H-index of 51. Professor Kwak JinYoung’s team (26) applied convolutional neural network(CNN) to distinguish between benign and malignant TN with diameters less than 10 mm, demonstrating diagnostic performance superior to that of ultrasound physicians. Professor Weixi and his team (27), whose publications ranked third in publication, developed a deep learning-based surface-enhanced Raman scattering chip to perform preoperative diagnosis of papillary thyroid carcinoma histological subtypes using thyroid fine needle aspiration specimens, while assessing lymph node metastasis. This method highlights the potential of personalized treatment, aiding in the formulation of individualized treatment strategies, reducing overtreatment, and lowering the risk of recurrence. Authors across different countries, particularly those based in China and the United States, can strengthen collaborative partnerships to drive forward the development of this research field. Researchers focusing on AI for preoperative diagnosis of TN can engage with prolific authors and research groups to access more resources and experience, thereby promoting further progress in the field.

The top ten high-output journals account for 29.85% of the total publication volume, highlighting their academic influence in the field. *Frontiers in Endocrinology* (58 publications), *Frontiers in Oncology* (50 publications), *Scientific Reports* (41 publications) and *Ultrasound in Medicine and Biology* published more than 30 publications. *Thyroid*, the official journal of the American Thyroid Association, with an impact factor of 6.6, making it the highest among high-output journals. These will assist researchers in selecting appropriate journals for the submission of relevant manuscripts. Additionally, researchers can follow these journals to get updates on relevant research. The three main citation paths revealed by the journal dual-map overlay analysis indicate that current research is primarily published in clinical medicine and imaging journals, while the research foundation is broadly derived from disciplines such as molecular biology, genetics, health medicine, and computational sciences. This interdisciplinary citation pattern shows the research as gradually transformed from basic research to clinical research, reflecting the diverse, integrative nature of research in this field and the trend of disciplinary convergence. This is the current concern of researchers.

High-frequency keywords such as “TN” and “deep learning” reflect the increasing integration of AI into research and daily clinical applications related to TN. Emergent keywords such as “CNN” and “semantic segmentation”, which appeared in 2017 and 2023, have continued to emerge, are important technologies in AI. “CNN”, as an emerging machine learning method, are widely used in the field of medical image classification. The workflow of CNN involves processing the lowest-level pixel information of input image matrices and progressively extracting important features layer by layer (28). Yu et al. (29) developed a multi-instance learning convolutional neural network model based on ultrasound features for the automatic identification and risk assessment of TN. Yang et al. (30) proposed a novel diagnostic model (CI-DUF) that integrates cytology and ultrasound image features through multimodal feature fusion and attention mechanism design to address the classification problem in FNAC. Semantic segmentation is a pixel-wise medical image analysis technique that assigns semantic labels to each pixel to distinguish lesions, normal parenchyma and background (31). Xing et al. (32) proposed a real-time semantic segmentation architecture termed fully convolutional dense dilated network (FCDDN) for TN segmentation on ultrasound images. The model ran fast with a few parameters and was suitable for medical devices that require real-time segmentation. Yu et al. (33) designed a weakly supervised segmentation framework named FBN to conduct pixel-level classification for TN ultrasound scans. Experiments showed that the model outperforms conventional approaches in segmentation accuracy and narrows the performance gap between benign and malignant lesions. This indicates that with the rapid development of AI technologies, algorithms in the preoperative diagnosis of TN are continuously making breakthroughs, significantly advancing the progress of auxiliary diagnostic systems in terms of accuracy, generalization ability, and clinical applicability.

Dynamic keyword burst detection further elucidates the developmental trajectory and research gaps of AI applications in preoperative diagnosis of TN. The development of TN–assisted diagnosis has progressed through three distinct phases: traditional feature–based differential diagnosis, deep learning–driven computer-aided diagnosis, and the current integration of semantic segmentation with radiomics. Concurrently, the research emphasis has gradually shifted from binary benign-malignant classification toward pixel-level lesion quantification and fine-grained feature extraction. Keywords with top burst strength, including benign and classification, verified that benign-malignant binary discrimination has always been the fundamental clinical target of this field.

To address the diagnostic challenge posed by indeterminate TN, numerous researchers have developed diverse AI models leveraging whole-slide imaging to distinguish benign and malignant indeterminate TN. These models can improve diagnostic consistency and reduce inter-observer variability (6). However, current efforts remain constrained by limited, non-representative training datasets and insufficient prospective clinical validation. More prospective trials are required to verify the clinical practicability of AI systems, standardize intelligent diagnostic workflows, and fully unlock the clinical potential of AI in thyroid pathology.

The application of AI in the preoperative diagnosis of TN not only highlights the value of interdisciplinary collaboration driven by ‘medical needs and engineering technology implementation,’ but also lays a solid foundation for the translation of core technologies into clinical practice. Looking ahead, multi-modal fused convolutional neural networks, thyroid-adapted semantic segmentation models, and AI-aided diagnosis systems tailored for individualized treatment planning will emerge as core research avenues to accelerate the clinical translation of intelligent thyroid diagnostic technologies. Ultimately, this will help build an intelligent clinical support system covering diagnosis, treatment, and management, achieving ‘precision medicine and smart healthcare in the diagnosis and treatment of TN.

### 4.2 Limitations

Our study has several limitations. First, only the WoS and Scopus databases were selected, excluding other databases such as PubMed, Cochrane Library, and Google Scholar. It may result in the exclusion of part studies indexed in other databases, which affecting the comprehensiveness of our findings. Only original research articles in English were included, which may introduce selection bias and limit the generalizability of the findings, especially regarding research trends and contributions in non-English speaking regions. Accordingly, we recommend that researchers maintain up-to-date awareness of the latest academic publications, especially those published in non-English languages, to develop a comprehensive, current understanding of the research field.

Secondly, citation-based indicators may also be influenced by self-citation patterns, which could affect the apparent academic impact of certain countries, institutions, or authors. Country attribution and the standardization of author and institutional name variants are limited by the accuracy of existing algorithms, which may introduce potential matching errors. However, given the large sample size and extended study period, these methodological limitations are unlikely to substantially change the overall research trends or the main conclusions of this study.

Furthermore, older publications may have an inherent citation advantage due to longer follow-up periods, which should be considered when interpreting citation-based rankings. The Data available for 2026 are incomplete, and recent citation indicators are affected by citation lag, which may underestimate the impact of newly published articles.

Finally, due to the rapid development of AI, related research may undergo significant updates within a short period. This study reflects the status of AI in the preoperative diagnosis of TN at a specific point in time, rather than the entire development process, and therefore, continuous data updates are necessary.

### 4.3 Conclusion

Since 2019, the application of AI in the preoperative diagnosis of TN has attracted increasing research interest, as reflected by the rapid growth in related publications. Chinese and American scholars have published a large number of articles in this field, and interdisciplinary collaboration has continued to expand. The research focus has gradually shifted from conventional diagnostic approaches toward pixel-level lesion quantification, fine-grained feature extraction and the integration of AI-enabled diagnostic approaches, highlighting the potential of AI to improve TN assessment. However, AI-assisted TN diagnosis remains primarily at the stages of model development, retrospective validation, and early clinical evaluation. Its translation into routine clinical practice requires further evidence from large-scale, multicenter, prospective studies, along with comprehensive evaluations of model robustness, interpretability, and real-world clinical benefits. Future research directions may include the development of deep learning algorithms to reduce operator-dependent variability, the construction of multimodal data fusion models, and improving clinical acceptance and trust in AI-assisted preoperative diagnosis of TN.

## Data Availability

The original contributions presented in the study are included in the article/supplementary material. Further inquiries can be directed to the corresponding author.

## References

[1] Durante C Grani G Lamartina L Filetti S Mandel SJ Cooper DS . The diagnosis and management of thyroid nodules: a review. Jama. (2018) 319:914–24. doi: 10.1001/jama.2018.0898 29509871

[2] Filho AM Laversanne M Ferlay J Colombet M Piñeros M Znaor A . The GLOBOCAN 2022 cancer estimates: data sources, methods, and a snapshot of the cancer burden worldwide. Int J Cancer. (2025) 156:1336–46. doi: 10.1002/ijc.35278 39688499

[3] Detweiler K Elfenbein DM Mayers D . Evaluation of thyroid nodules. Surg Clin North Am. (2019) 99:571–86. doi: 10.1016/j.suc.2019.04.001 31255192

[4] Grani G Sponziello M Filetti S Durante C . Thyroid nodules: diagnosis and management. Nat Rev Endocrinol. (2024) 20:715–28. doi: 10.1038/s41574-024-01025-4 39152228

[5] Peng S Liu Y Lv W Liu L Zhou Q Yang H . Deep learning-based artificial intelligence model to assist thyroid nodule diagnosis and management: a multicentre diagnostic study. Lancet Digit Health. (2021) 3:e250–9. doi: 10.1016/s2589-7500(21)00041-8 33766289

[6] Fiorentino V Pizzimenti C FranChina M Micali MG Russotto F Pepe L . The minefield of indeterminate thyroid nodules: could artificial intelligence be a suitable diagnostic tool? Diagn Histopathol. (2023) 29:396–401. doi: 10.1016/j.mpdhp.2023.06.013 38826717

[7] Sheffield BS Masoudi H Walker B Wiseman SM . Preoperative diagnosis of thyroid nodules using the Bethesda System for Reporting Thyroid Cytopathology: a comprehensive review and meta-analysis. Expert Rev Endocrinol Metab. (2014) 9:97–110. doi: 10.1586/17446651.2014.887435 30743753

[8] Rossi ED Tralongo P Fiorentino V Curatolo M Bruno C De Crea C . Approach to FNA of thyroid gland cysts. Adv Anat Pathol. (2022) 29:358–64. doi: 10.1097/pap.0000000000000357 35918293

[9] Cao CL Li QL Tong J Shi LN Li WX Xu Y . Artificial intelligence in thyroid ultrasound. Front Oncol. (2023) 13:1060702. doi: 10.3389/fonc.2023.1060702 37251934 PMC10213248

[10] Li Y Liu Y Xiao J Yan L Yang Z Li X . Clinical value of artificial intelligence in thyroid ultrasound: a prospective study from the real world. Eur Radiol. (2023) 33:4513–23. doi: 10.1007/s00330-022-09378-y 36622410

[11] Lee Y Alam MR Park H Yim K Seo KJ Hwang G . Improved diagnostic accuracy of thyroid fine-needle aspiration cytology with artificial intelligence technology. Thyroid. (2024) 34:723–34. doi: 10.1089/thy.2023.0384 38874262

[12] Page MJ McKenzie JE Bossuyt PM Boutron I Hoffmann TC Mulrow CD . The PRISMA 2020 statement: an updated guideline for reporting systematic reviews. Bmj. (2021) 372:n71. doi: 10.31222/osf.io/v7gm2_v1 33782057 PMC8005924

[13] Rethlefsen ML Kirtley S Waffenschmidt S Ayala AP Moher D Page MJ . PRISMA-S: an extension to the PRISMA statement for reporting literature searches in systematic reviews. Syst Rev. (2021) 10:39. doi: 10.1186/s13643-020-01542-z 33499930 PMC7839230

[14] Ninkov A Frank JR Maggio LA . Bibliometrics: methods for studying academic publishing. Perspect Med Educ. (2022) 11:173–6. doi: 10.1007/s40037-021-00695-4 34914027 PMC9240160

[15] Wang L Liu X Zhang K Liu Z Yi Q Jiang J . A bibliometric analysis and review of recent researches on Piezo (2010-2020). Channels (Austin). (2021) 15:310–21. doi: 10.1080/19336950.2021.1893453 33722169 PMC7971259

[16] Kim S Yeganova L Wilbur WJ . Meshable: searching PubMed abstracts by utilizing MeSH and MeSH-derived topical terms. Bioinformatics. (2016) 32:3044–6. doi: 10.1093/bioinformatics/btw331 27288493 PMC5039918

[17] Schreiber WE Giustini DM . Measuring scientific impact with the h-index: a primer for pathologists. Am J Clin Pathol. (2019) 151:286–91. doi: 10.1093/ajcp/aqy137 30346467

[18] Thompson DF Walker CK . A descriptive and historical review of bibliometrics with applications to medical sciences. Pharmacotherapy. (2015) 35:551–9. doi: 10.1002/phar.1586 25940769

[19] Chen CM Leydesdorff L . Patterns of connections and movements in dual-map overlays: a new method of publication portfolio analysis. J Assoc Inf Sci Technol. (2014) 65:334–51. doi: 10.1002/asi.22968 41531421

[20] Li X Zhang S Zhang Q Wei X Pan Y Zhao J . Diagnosis of thyroid cancer using deep convolutional neural network models applied to sonographic images: a retrospective, multicohort, diagnostic study. Lancet Oncol. (2019) 20:193–201. doi: 10.1016/s1470-2045(18)30762-9 30583848 PMC7083202

[21] Chi J Walia E Babyn P Wang J Groot G Eramian M . Thyroid nodule classification in ultrasound images by fine-tuning deep convolutional neural network. J Digit Imaging. (2017) 30:477–86. doi: 10.1007/s10278-017-9997-y 28695342 PMC5537102

[22] Patel KN Angell TE Babiarz J Barth NM Blevins T Duh QY . Performance of a genomic sequencing classifier for the preoperative diagnosis of cytologically indeterminate thyroid nodules. JAMA Surg. (2018) 153:817–24. doi: 10.1001/jamasurg.2018.1153 29799911 PMC6583881

[23] Chen CM . Science mapping: a systematic review of the literature. J Data Info Sci. (2017) 2:1–40. doi: 10.1515/jdis-2017-0006 31755547

[24] Li R Yang Y Wu S Huang K Chen W Liu Y . Using artificial intelligence to improve medical services in China. Ann Transl Med. (2020) 8:711. doi: 10.21037/atm.2019.11.108 32617331 PMC7327308

[25] Yao J Wang Y Lei Z Wang K Feng N Dong F . Multimodal GPT model for assisting thyroid nodule diagnosis and management. NPJ Digit Med. (2025) 8:245. doi: 10.1038/s41746-025-01652-9 40319170 PMC12049458

[26] Rho M Chun SH Lee E Lee HS Yoon JH Park VY . Diagnosis of thyroid micronodules on ultrasound using a deep convolutional neural network. Sci Rep. (2023) 13:7231. doi: 10.1038/s41598-023-34459-3 37142760 PMC10160046

[27] Hou ZK Zhao J Zhang M Hou W Li Y Yang Y . Preoperative identification of papillary thyroid carcinoma subtypes and lymph node metastasis via deep learning-assisted surface-enhanced Raman spectroscopy. ACS Nano. (2025) 19:21807–19. doi: 10.1021/acsnano.5c05698 40464771

[28] Chen C Mat Isa NA Liu X . A review of convolutional neural network based methods for medical image classification. Comput Biol Med. (2025) 185:109507. doi: 10.1016/j.compbiomed.2024.109507 39631108

[29] Yu D Song T Yu Y Zhang H Gao F Wang Z . Risk assessment of thyroid nodules with a multi-instance convolutional neural network. Front Oncol. (2025) 15:1608963. doi: 10.3389/fonc.2025.1608963 40777113 PMC12330392

[30] Yang D Li T Li L Chen S Li X . Multi-modal convolutional neural network-based thyroid cytology classification and diagnosis. Hum Pathol. (2025) 161:105868. doi: 10.1016/j.humpath.2025.105868 40617519

[31] Rickman J Struyk G Simpson B Byun BC Papanikolopoulos N . The growing role for semantic segmentation in urology. Eur Urol Focus. (2021) 7:692–5. doi: 10.1016/j.euf.2021.07.017 34417153

[32] Xing G Wang S Gao J Li X . Real-time reliable semantic segmentation of thyroid nodules in ultrasound images. Phys Med Biol. (2024) 69:025016. doi: 10.1088/1361-6560/ad1210 38048630

[33] Yu R Yan S Gao J Zhao M Fu X Yan Y . FBN: weakly supervised thyroid nodule segmentation optimized by online foreground and background. Ultrasound Med Biol. (2023) 49:1940–50. doi: 10.1016/j.ultrasmedbio.2023.04.009 37308370

